# Clinical Features and Survival Outcome of Early-Stage Primary Pulmonary MALT Lymphoma After Surgical Treatment

**DOI:** 10.3389/fsurg.2021.713748

**Published:** 2021-08-04

**Authors:** Ye Xu, Min Zheng, Qingkui Guo, Ning Wang, Rui Wang

**Affiliations:** ^1^Department of Thoracic Surgery, Tongren Hospital, Shanghai Jiao Tong University School of Medicine, Shanghai, China; ^2^Department of Thoracic Surgery, Shanghai Chest Hospital, Shanghai Jiao Tong University, Shanghai, China

**Keywords:** surgical treatment, prognosis, MALT, pulmonary, thoracic disease

## Abstract

**Background:** We aimed to study the clinical features and survival outcomes of patients with early-stage primary pulmonary mucosa-associated **lymphoid** tissue (MALT) lymphoma who underwent surgery.

**Methods:** This is a retrospective, single-center study including 32 patients with early-stage primary pulmonary MALT lymphoma. Univariate and multivariate Cox analyses were performed to select independent prognostic factors. The overall survival (OS) was analyzed by the Kaplan-Meier method and was compared with the log-rank test.

**Results:** Among the 32 patients included, there were 16 men (50.0%) and 16 women (50.0%). The average age was 59.2 years old. Ten patients had non-specific clinical symptoms including cough, expectoration, and chest pain, and four patients had B symptoms. CT images are not specific and can be shown as peripheral, central, solid, and ground glass but more peripheral (93.8%) and solid (75.0%). In prognostic analysis, univariate analysis showed that tumor stage and size were associated with relapse-free survival (RFS) and OS [hazard ratio (HR) = 1.105, 95% CI: 1.021–1.197, *P* = 0.011; HR = 1.211, 95% CI: 1.158–1.968, *P* = 0.003, respectively]. It seems to indicate that higher stage and larger size indicate a worse prognosis, but we could not find statistically significant predictors in multivariate analysis. Sublobectomy was performed in 21 (65.6) cases, lobectomy was performed in the other 11 (34.4) cases, both of them can achieve good prognosis (5-year RFS and OS are both 100%), and there is no significant difference between them.

**Conclusions:** The clinical manifestation of early-stage primary pulmonary MALT lymphoma is not significantly specific, and surgical resection is an effective treatment.

## Introduction

Mucosa-associated lymphoid tissue (MALT) lymphoma is the most common type of indolent B-cell primary pulmonary lymphoma, which originates from post-germinal center memory B cells (1). Primary pulmonary MALT lymphoma is a rare disease, also known as bronchial-associated lymphoid tissue lymphoma, accounting for 0.5% of all primary lung cancer (2, 3).

In nearly half of the MALT lymphoma cases, the patient is diagnosed with no symptoms or only some non-specific symptoms, including cough, mild dyspnea, and chest pain (4). CT images of pulmonary MALT lymphoma usually show no special findings, usually with chronic localized alveolar opacity <5 cm in diameter, and nearly 50% of the cases are accompanied by bronchial inflation sign (5–9). The etiology of primary pulmonary MALT lymphoma is not clear, and infection was considered to be the possible cause of MALT lymphoma. For example, the association between gastric MALT lymphoma and Helicobacter pylori is a well-known example. Recently, a European research team used a method based on the 16S ribosomal RNA (rRNA) gene and discovered Achromobacter xylosoxidans (A. xylosoxidans) in eight of nine lung MALT lymphomas. A. xylosoxidans is a gram-negative β-proteobacteria with low virulence but high resistance to antibiotic treatment. They further analyzed 124 cases of lung MALT lymphoma and 82 control tissues from six European countries. Overall, 57/124 (46%) lung MALT lymphomas and 15/82 (18%) control tissues were positive for A. xylosoxidans, and this suggests that MALT lymphoma of the lung may also be associated with infection (10). Primary pulmonary MALT lymphoma has a good prognosis with a 5-year survival rate of more than 85%. The treatment methods are different, such as surgery, radiotherapy, chemotherapy, and observation, but the results of surgical treatment of early-stage primary pulmonary MALT lymphoma are relatively few. In this study, we retrospectively analyzed the clinicopathological data of 32 patients with primary pulmonary MALT lymphoma treated by surgery to investigate the present clinical features of early-stage primary pulmonary MALT lymphoma and survival outcomes after surgical treatment.

## Patients and Methods

The institutional review board of **Tongren Hospital** supported this study. Patients with primary pulmonary MALT lymphoma who underwent surgical resection between 2009 and 2015 were all identified consecutively. The diagnosis of MALT lymphoma was based on the criteria of WHO. In morphology, the tumor cells have small-sized and medium-sized lymphocytes, the nucleus is slightly irregular, the nucleolus is not obvious, similar to the central cell, and the cytoplasm is relatively rich and pale. Immunohistochemical staining showed positive expressions of CD20 and CD79a. Immunohistochemical study of cluster of differentiation (CD)5, CD10, CD23, cyclin D1, and Ki-67 was performed in patients with low-grade B-cell lymphoma that was difficult to rule out. Fluorescence *in situ* hybridization (FISH) was used to detect MALT-1 gene rearrangement in pathological diagnosis. All diagnoses were performed by two pathologists.

The lesions were staged according to the modified Ann Arbor classification (11). At the same time, we also provide the staging criteria as Supplementary Table 1. All the patients received preoperative assessments, such as chest CT scanning, abdominal CT or ultrasound, brain CT scanning or MRI, and radionuclide bone scanning, to confirm the diagnosis of primary lung tumor. All patients received a routine peripheral blood examination before surgery, and abnormal count or morphology of red blood cells, white blood cells, and platelets was recorded. Finally, a total of 32 patients with primary pulmonary MALT lymphoma were included in the study. All 32 patients had R0 resection, and none of them received adjuvant therapy after surgery. The principle of follow-up was that the patients were followed up by outpatient clinic or telephone every 3 months in the first year, one time every 6 months in the following 3 years, and then one time a year.

## Statistical Analysis

All the clinicopathological data and distributions of survival were analyzed by Statistical Package for the Social Sciences (SPSS) 23.0 software package (SPSS Inc., Chicago, IL, USA) and Prism 5 (Graphpad Software Inc., La Jolla, CA, USA). The curves of relapse-free survival (RFS) and overall survival (OS), as well as the comparisons, were calculated by Kaplan-Meier survival curves and the log-rank test. Univariate and multivariate Cox analyses were performed to select independent prognostic factors. Two-sided *P* < 0.05 was set as statistical significance in this study.

## Results

### Clinical Features

The characteristics are all listed in Table 1. Of those 32 patients included in the study, there were 16 men (50.0%) and 16 women (50.0%), with an average age of 59.2 ± 10.0 years old. A total of 22 patients showed no symptoms, while respiratory symptoms, such as cough, dyspnea, sputum, and chest pain, were the most common in other patients, accounting for 31.2% of the total population. B symptoms including fever of unknown origin (above 38°C), weight loss (more than 10% of body weight within 6 months), and night sweats occurred in four cases. In clinical staging, according to the Ann Arbor staging method, 24 (75%) patients were diagnosed as stage I, and the remaining 8 (25%) patients were diagnosed as stage II (all were stage IIE). Among the 32 patients, lobectomy was performed in 21 (65.6) cases, and sublobectomy was performed in the other 11 (34.4) cases (including seven wedge resections and four segmentectomies). A total of 22 patients underwent systemic lymphadenectomy, five patients underwent sampled lymphadenectomy, and the remaining five patients did not undergo lymphadenectomy. No one received adjuvant treatment after surgery.

**Table 1:** Clinical characteristics of patients with primary pulmonary mucosa-associated **lymphoid** tissue (MALT) lymphoma.

**Characteristic**	**No. (%)**
**Total:** ***n*** **=** **32**	
Age	
Mean ± SD	59.2 ± 10.0
Gender	
Male	16 (50.0)
Female	16 (50.0)
Smoking status	
No	25 (78.1)
Yes	7 (21.9)
Preoperative trilineage hematopoiesis abnormality	
No	30 (93.8)
Yes	2 (6.2)
Symptom	
No	22 (68.8)
Yes	10 (31.2)
Symptom B	
No	28 (87.5)
Yes	4 (12.5)
Stage	
I	24 (75.0)
II	8 (25.0)
Surgery type	
Lobectomy	11 (34.4)
Sublobectomy	21 (65.6)
Lymph node dissection
Systematically	22 (68.8)
Sampling	5 (15.6)
None	5 (15.6)
CT image expression	
Solid nodule	24 (75.0)
GGO	8 (25.0)
Location	
Peripheral	30 (93.8)
Central	2 (6.2)
Tumor Size	
Mean ± SD	2.4 ± 1.8
Blood type	
A	9 (28.1)
B	8 (25.0)
AB	9 (28.1)
O	6 (18.8)

**Figure 1:**
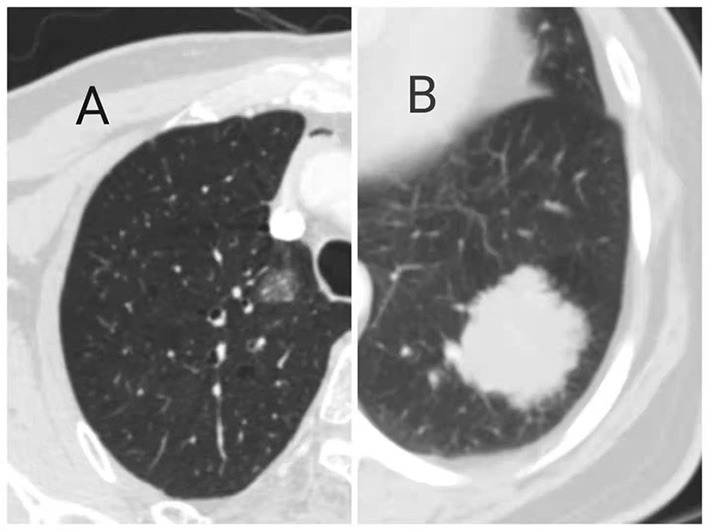
Primary pulmonary mucosa-associated **lymphoid** tissue (MALT) lymphoma showed ground glass **(A)** and solid **(B)** on CT imaging.

**Figure 2:**
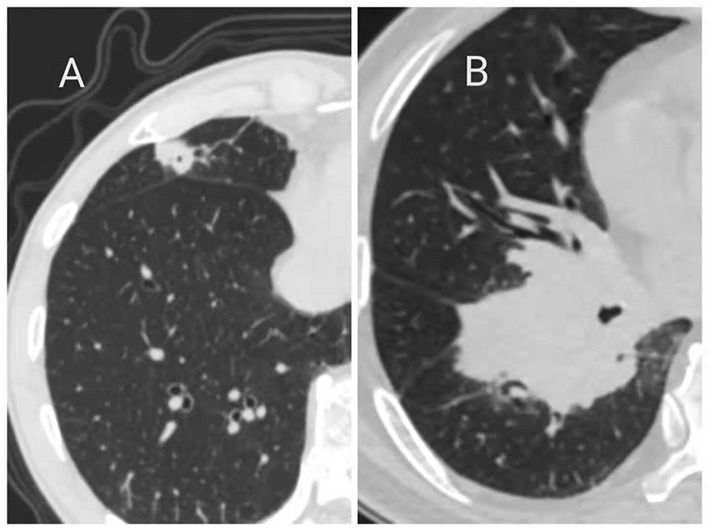
Primary pulmonary MALT lymphoma showed peripheral **(A)** and central **(B)** types on CT Imaging.

In terms of imaging findings, 24 (75%) patients showed solid lesions and the other 8 (25%) patients showed ground-glass lesions (Figure 1). A total of 21 (65.6%) lesions were located in the right lung, including six in the right upper lobe, seven in the right middle lobe, eight in the right lower lobe, and 11 in the left lung, including eight in the left upper lobe and three in the left lower lobe. The lesions distal to the segmental bronchi are defined as the peripheral type and above the segmental bronchi are defined as the central type; on this basis, 30 (93.8) cases were peripheral type and 2 (6.2) cases were central type (Figure 2).

### Survival Analysis

**Figure 3:**
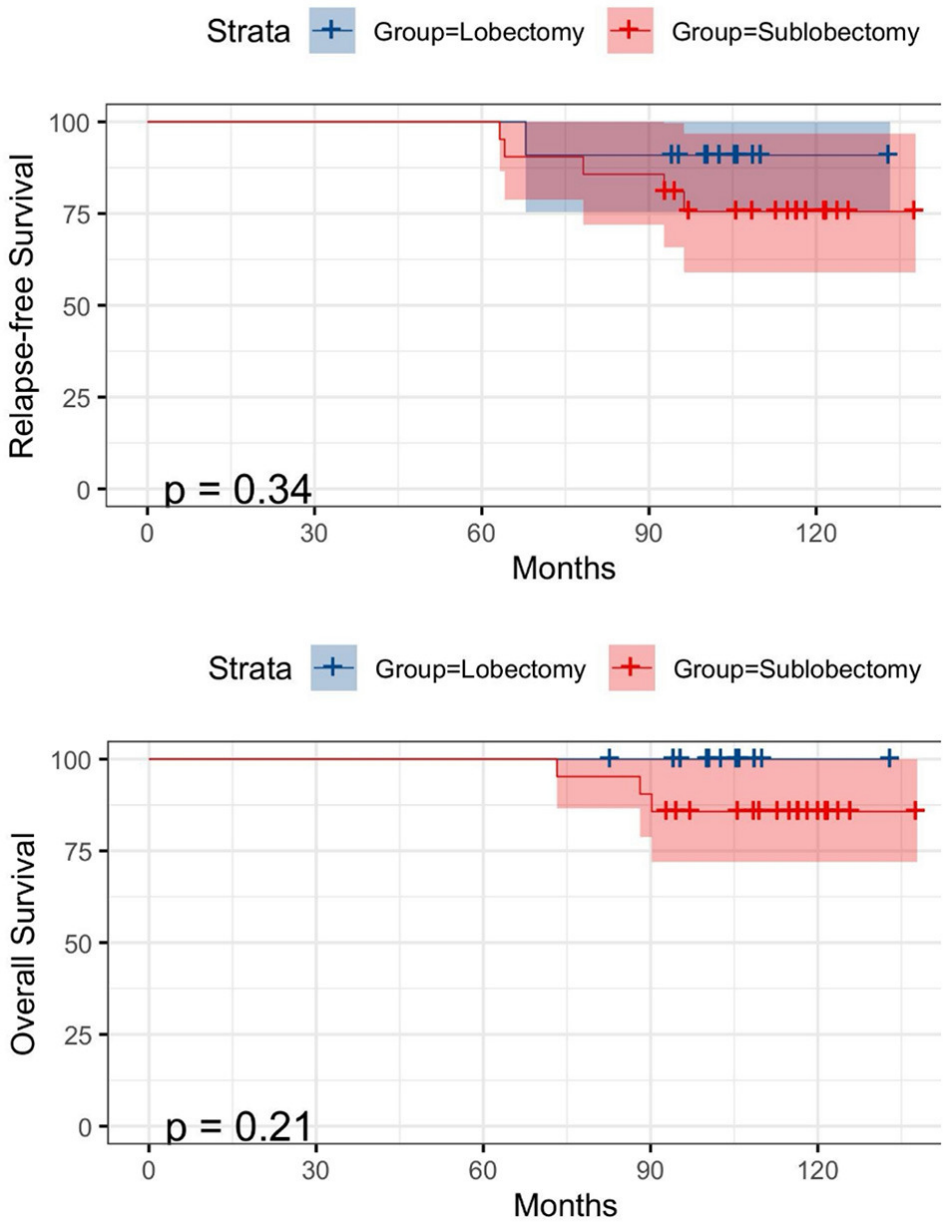
Kaplan-Meier survival curves for relapse-free survival (RFS) (above) and overall survival (OS) (below) according to lobectomy and sublobectomy.

**Figure 4:**
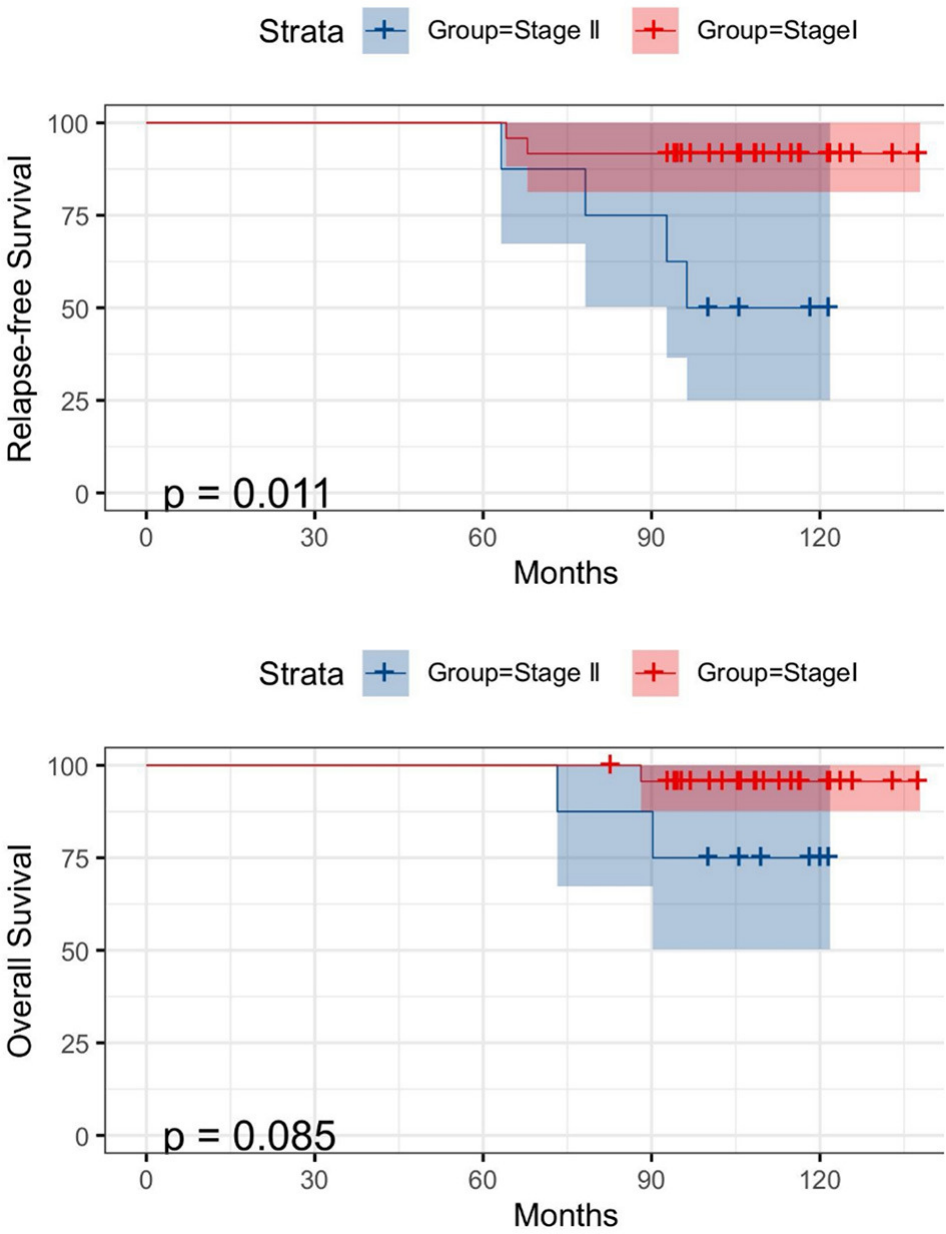
Kaplan-Meier survival curves for RFS (above) and OS (below) according to tumor stage.

During the follow-up, six of the 30 patients experienced a relapse, and finally three patients died (disease-related). The median follow-up time and the median survival time of these patients were 98.3 and 96.4 months, respectively. Figure 3 shows the RFS and OS comparisons between lobectomy and sublobectomy groups. Although, the analysis showed that lobectomy had a better prognosis, there was no significant difference between the two groups. Univariable analysis revealed that both tumor stage (HR = 1.105, 95% CI: 1.021–1.197, *P* = 0.011) and size (HR = 1.211, 95% CI: 1.158–1.968, *P* = 0.003) were significant predictors of RFS and that only tumor size (HR = 1.714, 95% CI: 1.558–1.968, *P* = 0.023) was a significant predictor of OS, while no significant predictor was found in multivariable analysis (Tables 2, 3). The log-rank analysis identified tumor stage (Figure 4) as factors associated with RFS but not with OS.

**Table 2:** Univariable and multivariable analyses for relapse-free survival (RFS) in patients with resected primary pulmonary MALT lymphoma.

	**Univariable**			**Multivariable**		
	***P***	**HR**	**95% CI**	***P***	**HR**	**95% CI**
Age	0.063	1.247	0.672–1.423			
Gender	0.237	1.236	0.725–1.324			
Smoking status	0.365	0.899	0.637–1.267			
Preoperative trilineage hematopoiesis abnormality	0.729	0.830	0.315–3.918			
Symptom	0.424	1.139	0.323–1.206			
Symptom B	0.089	1.217	0.608–1.097			
Stage	**0.011**	1.105	1.021–1.197	0.225	1.055	0.967–1.151
Surgery type	0.686	1.399	0.169–1.675			
Lymph node dissection	0.675	1.135	0.554–1.253			
CT image expression	0.765	0.875	0.341–1.692			
Tumor Size	**0.003**	1.211	1.158–1.968	0.356	1.274	1.125–1.621
Blood type	0.738	0.736	0.551–1.983			

**Table 3:** Univariable and multivariable analyses for overall survival (OS) in patients with resected primary pulmonary MALT lymphoma.

	**Univariable**			**Multivariable**		
	***P***	**HR**	**95% CI**	***P***	**HR**	**95% CI**
Age	0.072	1.325	0.823–1.383			
Gender	0.315	1.315	0.843–1.245			
Smoking status	0.765	0.823	0.937–1.123			
Preoperative trilineage hematopoiesis abnormality	0.822	0.933	0.815–3.766			
Symptom	0.624	1.139	0.323–1.206			
Symptom B	0.119	1.417	0.912–1.453			
Stage	0.085	1.235	1.321–1.457			
Surgery type	0.214	1.256	0.356–2.578			
Lymph node dissection	0.765	1.211	0.897–1.564			
CT image expression	0.879	0.876	0.756–1.992			
Tumor Size	**0.023**	1.714	1.558–1.968	0.356	1.234	0.926–1.621
Blood type	0.738	0.736	0.551–1.983			

## Discussion

Primary pulmonary MALT lymphoma is a rare disease that requires rigorous and rigorous diagnostic examination. In this study, we analyzed the clinical prognostic information of 32 patients with primary pulmonary MALT lymphoma diagnosed by postoperative pathology through a retrospective study. It was found that about 10 patients (31.2%) had clinical symptoms such as cough, expectoration, and chest pain before the operation, while only 4 (12.5%) patients had B symptoms. Our data are basically consistent with the results of the previous studies, which indicated that respiratory symptoms presented in 55–63% patients and B symptoms presented in 15–22% patients (12–15). However, the clinical symptoms are not specific, so it is difficult for us to make a diagnosis based on these clinical symptoms alone.

The manifestation of primary pulmonary MALT lymphoma on CT images is also not specific. CT images of the lesions are varied, which can be solid or ground glass-like and can occur in different lung lobes. However, most of the lesions turned into peripheral type. The previous studies have shown that most MALT lesions are bilateral (60–70% of cases) and multiple (70–77% of cases) and that there is no predilection for location (16, 17). The most common types are consolidation (55%), nodules (55%), and masses (50%). About 85% of the patients have airways. Small nodules (20%), ground-glass shadows (25%), and septum (10%) are less common (16–18). Finally, the CT scan mode may be solitary pulmonary nodules, and more rare are cystic or cavitary lesions.

Patients with MALT lymphoma had a good prognosis, with an overall 5-year survival rate of more than 80% and a median survival time of more than 10 years (5–9, 18). The previous studies have shown that there are differences in the prognosis of MALT lymphoma in different sites: The median survival time of patients with MALT lymphoma in the digestive tract is not different from that in other sites, but the progression-free survival time in other parts of the disease, especially lung disease, seems to be shorter (2).

The prognostic factors of MALT lymphoma are not clear. Sex, delayed diagnosis, presence of symptoms, bilateral lesions, extrapulmonary involvement, or medulla location could not predict the prognosis (13). In multivariate analysis, including all lesion sites, elevated β-2-microglobulin levels, and stage IV classification according to the Ann Arbor system were found to affect prognosis. In a study of 63 patients with pulmonary MALT lymphoma, age, and performance were adverse prognostic factors affecting OS (13). Another retrospective study involving 48 patients with MALT lymphoma did not find any prognostic factors at all (11). In a prognostic study of 180 patients with lung lymphoma (67% MALT), Zhang et al. (19) found that none of the prognostic factors they studied significantly affected survival in indolent lung lymphoma, which is consistent with our findings. Recently, Thieblemont et al. (20) have shown that age >70 years, Ann Arbor stage >2, and elevated lactate dehydrogenase are simple and effective prognostic factors. It should be noted that the objective of this study is not limited to primary pulmonary MALT lymphoma. In a group of 393 patients, these three factors were used to distinguish between the three risk groups. The 5-year progression-free survival rates were 78, 63, and 29%, respectively (*P* < 0.001), and the 5-year overall survival rates were 99, 92, and 74%, respectively (*P* < 0.001) (21). In our study, all our cases were at an early stage, and univariable analysis revealed that only tumor stage and size were significant predictors of RFS and OS, while no significant predictor was found in multivariable analysis. Although, our results failed to find effective predictors, patients with higher tumor size and stage tended to have a poor prognosis. Our results also show that the extent of surgical resection has little effect on the prognosis.

There are several limitations to this study. First, the sample size of primary pulmonary MALT lymphoma was relatively small, so there may produce a bias. Second, those clinical and pathological data were retrospective in nature, and they should be confirmed in prospective trials if possible.

In conclusion, primary pulmonary MALT lymphoma is an indolent disease with favorable treatment outcomes. Although, no significant independent prognostic factors were found in multivariate analysis, the prognosis may be poor for tumors with larger size and higher stage. Surgery still plays an important role in the diagnosis and treatment of early-stage primary pulmonary MALT lymphoma.

## Data Availability Statement

The raw data supporting the conclusions of this article will be made available by the authors, without undue reservation.

## Author Contributions

RW: conception, design, and administrative support. YX, MZ, and QG: provision of study materials or patients. YX, MZ, and NW: collection and assembly of data. YX and RW: data analysis and interpretation. All authors contributed to the article and approved the submitted version.

## Conflict of Interest

The authors declare that the research was conducted in the absence of any commercial or financial relationships that could be construed as a potential conflict of interest.

## Publisher's Note

All claims expressed in this article are solely those of the authors and do not necessarily represent those of their affiliated organizations, or those of the publisher, the editors and the reviewers. Any product that may be evaluated in this article, or claim that may be made by its manufacturer, is not guaranteed or endorsed by the publisher.
