# Supplementary material for: Clinical Features and Survival Outcome of Early-Stage Primary Pulmonary MALT Lymphoma After Surgical Treatment
Source: Front Surg. 2021 Aug 4;8:713748. doi: 10.3389/fsurg.2021.713748 (PMC8371471; doi:10.3389/fsurg.2021.713748)
Supplement: Supplementary file 1 [file Data_Sheet_1.DOCX]

The staging classification used for extranodal lymphomas is as follows:

Stage I E: Involvement of lung only (can be bilateral)

Stage II 1E: Lung and hilar lymph nodes

Stage II 2E: Lung and mediastinal lymph nodes

Stage II 2EW: Lung and adjacent chest wall or diaphragm

Stage III: Involvement of lung and of lymph nodes below diaphragm

Stage IV: Diffuse involvement of one or more extralymphatic organs or tissues
